# Combined cut down and endovascular retrieval of orphaned ventriculoatrial shunt with stenting of chronic superior vena cava occlusion

**DOI:** 10.1007/s00247-020-04943-3

**Published:** 2021-01-12

**Authors:** Prahasit Thirkateh, Ahsun Riaz, Matthew C. Tate, Seth Stein, Scott A. Resnick

**Affiliations:** 1 Chicago Medical School at Rosalind Franklin University of Medicine and Science, 3333 Green Bay Road, North Chicago, IL 60064 USA; 2 Department of Radiology, Northwestern University Feinberg School of Medicine, Chicago, IL USA; 3 Department of Neurological Surgery, Northwestern University Feinberg School of Medicine, Chicago, IL USA; 4 Department of Neurology, Northwestern University Feinberg School of Medicine, Chicago, IL USA; 5 Division of Interventional Radiology, Northwestern University Feinberg School of Medicine, Chicago, IL USA

**Keywords:** Adult, Interventional radiology, Revascularization, Stent, Superior vena cava, Superior vena cava syndrome

## Abstract

Revascularization of the superior vena cava (SVC) in the context of symptomatic luminal obstruction is a therapeutic intervention performed for SVC syndrome of benign or malignant etiology. Venous occlusion can preclude future access and cause symptoms ranging from mild chest discomfort to the more serious effects of SVC syndrome. This case report demonstrates the treatment of a novel case of SVC syndrome arising from a previously placed SVC stent. An intravascular, extraluminal orphaned ventriculoatrial shunt was used to go through the SVC but around the existing lumen-limiting stent to place a new larger stent for revascularization. This case highlights the need for an innovative approach for complex foreign body retrieval and treatment of chronic SVC occlusion.

## Introduction

Superior vena cava (SVC) obstruction can arise from a variety of etiologies including thoracic malignancies, thrombus from a retained intravascular device and fibrosing mediastinitis. Symptomatic obstruction of the SVC can result in face and neck swelling, dyspnea at rest, cough, upper extremity swelling, syncope, dizziness, hoarseness and dilated chest collateral veins; these constitute a process termed SVC syndrome [1, 2]. In cases with SVC syndrome arising from malignancy, therapy can involve surgical resection, chemotherapy and radiation therapy. Endovascular reconstruction with balloon angioplasty with or without stent placement is effective in achieving symptomatic relief of benign SVC syndrome, rendering it the current mainstay of treatment [3]. This report describes a case of SVC syndrome as a result of a diameter-limiting, long-standing, self-expanding stent placed in a child. This was initially deemed untreatable via endovascular means until an orphaned ventriculoatrial shunt catheter was noted traversing the SVC stenosis and positioned outside the existing diameter-limiting stent. This abandoned ventriculoatrial shunt catheter was used as an access route for subsequent SVC stent placement and revascularization.

## Case report

**Fig. 1:**
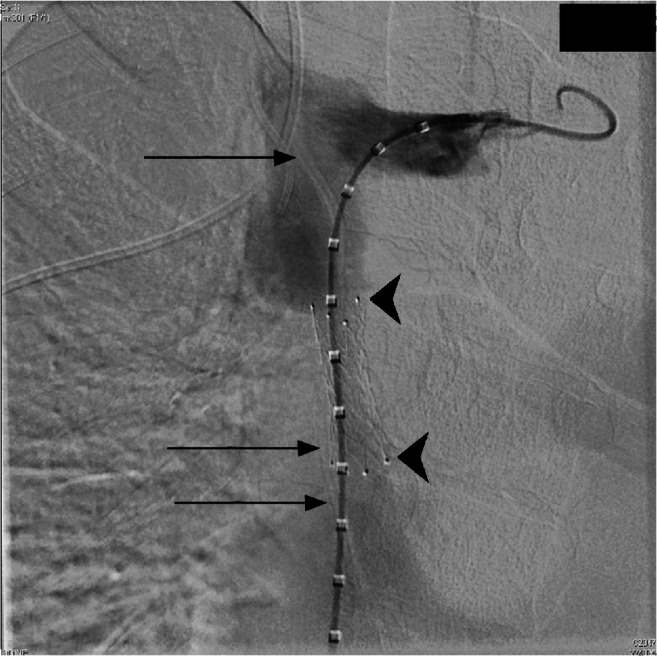
Anteroposterior view from preprocedural venogram shows the superior vena cava stenosis with decreased luminal size over the previously placed nitinol stent (*arrowheads*). Borders of the abandoned ventriculoatrial shunt are depicted by the *arrows*

A 22-year-old man with a past medical history of congenital renal dysplasia with end-stage renal disease, Chiari I malformation with hydrocephalus treated by a ventriculopleural shunt for drainage and chronic SVC stenosis presented with symptoms of SVC syndrome, including head, neck, face and upper extremity swelling with near syncope when leaning forward. The patient has a remote history of stent placement at the age of 8 for symptomatic SVC syndrome. The stent used was an 8-mm self-expanding nitinol (nickel-titanium) alloy stent, and despite previous balloon dilation of in-stent hyperplastic narrowing, SVC syndrome symptoms persisted, likely secondary to an inability to over-dilate the stent substantively beyond the nominal diameter. Radiographic imaging revealed a narrowed SVC luminal size of 7 mm along the 4-cm length of the nitinol stent (Fig. 1), as well as a corresponding abandoned (nonfunctioning) ventriculoatrial shunt catheter bridging the SVC length and located advantageously outside the existing stent. Further investigation revealed that the shunt was no longer being used for cerebrospinal fluid drainage as the patient had a working ventriculopleural shunt. The abandoned ventriculoatrial shunt catheter, the diameter of which was determined to be either 7 or 8 French (Fr), was found to be intact with cranial margin transected and free in the mid right internal jugular vein. As it was believed the indwelling nitinol stent could not durably be expanded beyond the nominal stent diameter due to nitinol stent construction techniques, a plan was made for SVC lesion traversal outside of the stent and placement of a high radial force large-diameter stent within the SVC lumen, displacing the undersized stent to the lumen periphery. The orphaned ventriculoatrial shunt catheter was to play a critical role in the procedure by allowing for traversal of the SVC lesion outside of the diameter-limited indwelling stent.

**Fig. 2:**
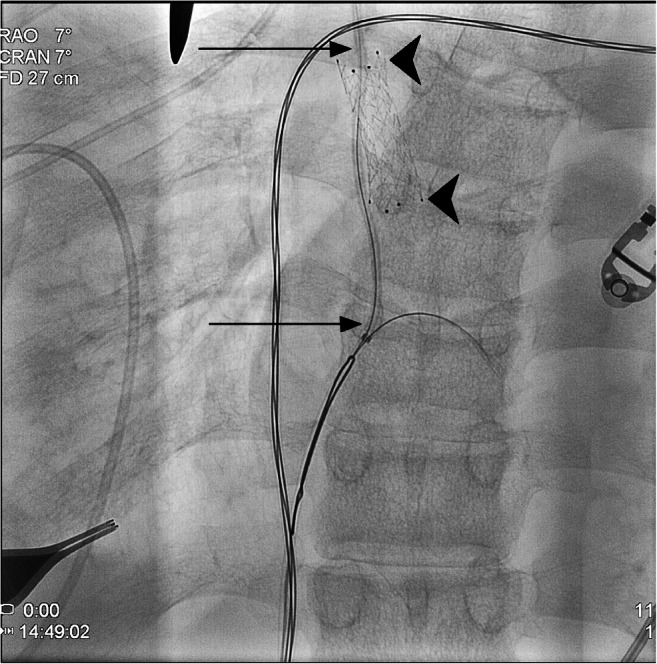
Anteroposterior view from intraprocedural venogram shows access of ventriculoatrial shunt catheter using a guidewire. Superior and inferior portions of the shunt catheter with an intraluminal guidewire in place are depicted by the upper and lower *arrows*. Upper and lower borders of the preexisting stent are depicted by the *arrowheads*

**Fig. 3:**
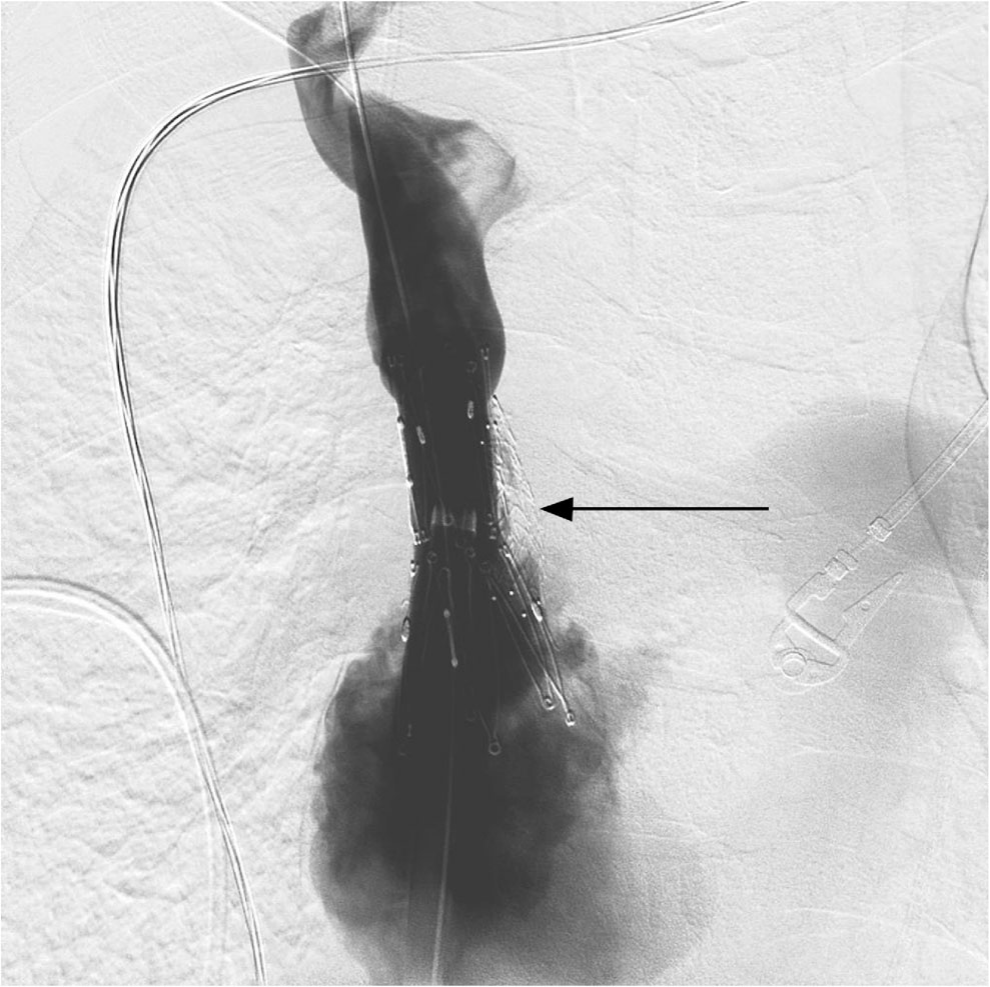
Anteroposterior view from final venogram shows postprocedural visualization of superior vena cava with the Z-stent in place and increased luminal patency (*arrow*)

An initial percutaneous attempt at ventriculoatrial shunt catheter access was unsuccessful as the catheter appeared to course within the carotid sheath in the lower neck where preferred catheter access was undertaken. Subsequently, with the patient under general anesthesia, our neurosurgery colleagues performed a jugular cutdown to access the proximal end of the orphaned ventriculoatrial shunt catheter. Femoral access was then obtained and a 0.014-in., 300-cm Fathom 14 guidewire (Boston Scientific, Marlborough, MA) passed through the catheter, thereby traversing the SVC lesion alongside the indwelling undersized stent, and into the right atrium (Fig. 2). The guidewire was then engaged with a 20-mm gooseneck snare device (Medtronic, Minneapolis, MN) and withdrawn via a previously placed 8-Fr, 55-cm common femoral vein Rabbe sheath (Cook Medical, Bloomington, IN) to create through-and-through wire access. The ventriculoatrial catheter fragment was then removed by withdrawing it slowly over the guidewire via the 8-Fr jugular access sheath, utilizing the femoral access sheath introducer to push the catheter cephelad. Using standard guidewire and catheter exchange technique the thru wire was exchanged for a 0.035-in., 300-cm Storq stiff guidewire (Cordis, Santa Clara, CA) via a 0.035-in., 90-cm QuickCross support catheter (Philips Medical, Stamford, CT). The femoral access sheath was exchanged for a 16-Fr, 70-cm sheath (Cook Medical). Through this access, a 6-mm, followed by a 10-mm (Mustang; Boston Scientific) and a 14-mm (Atlas; Bard Peripheral Vascular, Tempe, AZ) SVC balloon angioplasty (balloon 4 cm in length) adjacent to the existent stent was performed. Subsequently, the 16-Fr sheath was advanced across the SVC lesion and adjacent to the existing stent using the sheath introducer and a 25-mm × 5-cm Z configuration self-expanding stent (Cook Medical) deployed via this access bridging the SVC lesion and the indwelling stent, followed by an 18-mm (Atlas; Bard Peripheral Vascular) post stent deployment balloon angioplasty. Control venography was performed following each balloon inflation to assure SVC integrity. Wide patency of the SVC was seen following stent placement and final balloon angioplasty with the preexisting nitinol SVC stent displaced to the side and out of the flow channel (Fig. 3).

No significant complications were encountered during the procedure. Complete symptom resolution was noted following the procedure, but the patient did complain of a dull chest ache that improved over the subsequent 3-month follow-up period. Chest CT with venous phase imaging at a 3-month follow-up showed wide patency of the SVC.

## Discussion

Given this patient’s history of symptomatic SVC syndrome as a child and multiple SVC balloon angioplasty procedures without durability, a self-expanding nitinol stent was placed during childhood. Subsequently, the patient continued to grow, but the nitinol stent had reached its nominal size and could not be expanded further, resulting in a significant size mismatch between the stent diameter and the SVC diameter. The associated fixed SVC narrowing and flow limitation led to symptomatic SVC narrowing as an adult.

The nitinol stent used for the patient’s initial presentation with SVC syndrome is a viable option for vascular stenting in severe stenosis. Literature suggests that nitinol stents are the preferred option over standard metal stents due to their high flexibility, decreased thrombogenicity and high radial expansive force [4]. In the case of our patient, the initial nitinol stent was maximally expanded to nominal diameter via balloon angioplasty; however, this did not resolve the patient’s symptoms as the nominal stent diameter was inadequate for an adult SVC. The choice of a Z-configuration stent was made due to the high stent radial force when compared to the existing nitinol stent [5], allowing us to take advantage of the collapsibility of the nitinol stent with the deployment of the Z-stent in the same region. Given the patient’s age and the likely absence of additional growth of the SVC past the current size, the authors believe the Z-stent will allow for durable patency of the SVC with constant compression of the nitinol stent to the side vessel wall.

Post-procedural luminal diameter at 18 mm was found to be more than double the pre-procedural luminal diameter of 8 mm, resulting in a nearly fivefold increase in SVC cross-sectional area and subsequent marked increase in flow capacity.

This case presented two main challenges. First, foreign body retrieval often requires an innovative approach, balancing the risks of catheter migrations and vascular rupture. A multidisciplinary team may be necessary, as purely surgical or endovascular techniques may not be possible without the other. In this case, the cutdown was essential to provide through-and-through access, as the cranial aspect of the orphaned catheter was not patent and embedded in the soft tissues of the neck. This improved the safety of the procedure by allowing both percutaneous and endovascular access to a potentially damaged vessel. Ventriculoatrial shunt catheters have the ability to migrate to the pulmonary arteries, as reported by James et al. [6], and various tools and techniques can be employed for retrieval, including various loop snare devices, forceps, hooked catheters and balloon catheters. As mentioned by the authors, the gooseneck snare is relatively atraumatic and was successfully employed in this case to grasp the atrial end of the orphaned catheter.

The second challenge of this case was the treatment of SVC stenosis with an indwelling undersized stent placed during the patient’s childhood. A retrospective cohort study by the Pediatric Cardiac Care Consortium of patients with congenital cardiac disease demonstrated that young patients (average age: 11 years) who were treated with SVC stenting were 7 times less likely to undergo repeat intervention within 6 months than those treated with venoplasty alone, but had a slightly higher rate of late reintervention likely in part due to patient growth [7]. Tzifa et al. [8] studied patients who had SVC obstruction at Boston Children’s Hospital over a 22-year period and found that stents were more effective at treating the obstruction and, for patients older than 5 years, freedom from reintervention was higher in the stent group.

Stent sizing in the growing patient can lead to worsening venous stenosis or even stent migration as the patient ages. In this case, the venous stenosis recurred and the operators performed balloon venoplasty to re-expand the SVC lumen. This compressed the old stent and allowed for ample space to deploy an appropriately sized stent. The Z-stent was deployed, primarily due to its large luminal diameter, allowing for revascularization of the SVC.

Usage of a shunt catheter as a method for new stent deployment for revascularization was determined to be the most appropriate therapeutic option for this patient’s SVC stenosis given the previous unsuccessful attempts through balloon angioplasty. The primary, more invasive alternative to endovascular recanalization of such a case is surgical reconstruction using an autologous saphenous venous bypass graft to circumvent the area of stenosis. If recanalization using stenting had failed, the open surgical approach would have been the most appropriate next step in the care of this patient. A distant alternative could have been removal of the abandoned catheter and medical management with anticoagulation; however, given the severity of the patient’s symptoms and the corresponding degree of luminal stenosis, this may not have been adequate as a treatment option.
